# Acquired Hemophilia A Presenting as Massive Postoperative Bleeding in a Patient with Oral Squamous Cell Carcinoma

**DOI:** 10.1155/2020/8961785

**Published:** 2020-09-03

**Authors:** Susumu Oba, Mitsuhiko Nakahira, Yasunao Kogashiwa, Yasuhiro Ebihara, Masashi Sugasawa

**Affiliations:** ^1^Department of Otolaryngology and Head and Neck Surgery, Tokyo Metropolitan Police Hospital, 4-22-1 Nakano, Nakano, Tokyo 164-8541, Japan; ^2^Department of Head and Neck Surgery, Saitama Medical University International Medical Center, 1397-1 Yamane, Hidaka, Saitama 350-1298, Japan

## Abstract

Acquired hemophilia A (AHA) is an extremely rare and serious bleeding disorder caused by autoantibodies against coagulation factor VIII (FVIII). Approximately, 10% of patients with AHA have an underlying malignancy. We report on a 46-year-old man with AHA and advanced oral cancer who presented with massive bleeding after surgery. Preoperative blood coagulation tests showed no abnormalities. He underwent radical tumor resection followed by reconstruction using a free rectus abdominal musculocutaneous flap. Massive subcutaneous hemorrhage developed in his neck and abdomen on the first postoperative day. The hemorrhage remained uncontrolled, despite embolization of the responsible vessels. Subsequent laboratory data showed prolonged activated partial thromboplastin time and decreased FVIII levels. On the basis of his clinical course and the presence of the FVIII inhibitor, we speculated that the patient suffered from AHA. We administered recombinant activated factor VII and prednisolone, after which the spontaneous bleeding stopped and the subcutaneous hemorrhage resolved. A review of the literature identified only three previous documented cases of AHA associated with head and neck cancer. This case indicates that AHA should not be ruled out in patients with uncontrolled postoperative bleeding, while attempting to ensure bleeding control and preventing potentially catastrophic fatal consequences.

## 1. Introduction

Acquired hemophilia A (AHA) is a serious bleeding disorder caused by autoantibodies against coagulation factor VIII (FVIII). The typical clinical manifestation is sudden extensive bleeding, and the associated mortality ranges from 8% to 22% [1–6]. The incidence of AHA is extremely rare, with approximately 0.2–1.48 cases per million per year [1, 4, 7, 8]. Acquired FVIII inhibitors are classically associated with chronic inflammatory disorders, autoimmune disorders, pregnancy, malignancies, multiple transfusions, and drugs [1–5, 7, 8]. Approximately, 10% of patients with AHA have an underlying malignancy [9], and many have undergone surgery. Surgery has also recently been recognized as a possible etiological factor in bleeding episodes [5].

However, there is currently a lack of data regarding the clinical presentation and management of cancer patients with AHA. Moreover, the rarity and diversity of AHA mean that it has not been feasible to perform prospective randomized clinical trials to determine the most effective treatment protocols. A recent systematic literature review of AHA in cancer patients found that head and neck cancer accounted for only three of 60 cases of solid cancer associated with AHA [10]. In this case report, we describe the successful management of massive postoperative bleeding caused by AHA in a patient with oral squamous cell carcinoma.

## 2. Case Presentation

A 46-year-old man presented with a 4-month history of trismus and was diagnosed with buccal mucosal squamous cell carcinoma (T4aN2bM0). He had no personal or family history of spontaneous bleeding. His medical history was unremarkable, and he reported no known drug allergy. Preoperative blood coagulation tests were normal: prothrombin time-international normalized ratio (PT-INR), 1.10 (normal range: 0.85–1.28) and activated partial thromboplastin time (APTT), 31.2 s (normal range: 25.0–40.0 s).

One cycle of preoperative chemotherapy with docetaxel (60 mg/m^2^ on day 1) and cisplatin (75 mg/m^2^ on day 1) was performed for disease control (3 weeks' total therapy time), with no severe adverse events. Radical oral tumor resection was performed 4 weeks after the start of chemotherapy, including segmental mandibulotomy, modified maxillectomy, and neck dissection followed by reconstruction using a free rectus abdominal musculocutaneous flap under general anesthesia. This procedure required a long operation time (538 minutes) and resulted in extensive blood loss (1260 g), requiring 4450 mL intravenous fluid infusion and transfusion of 4 units of red blood cells during the operation. The procedure was ultimately completed successfully with no critical events associated with intractable bleeding.

**Figure 1:**
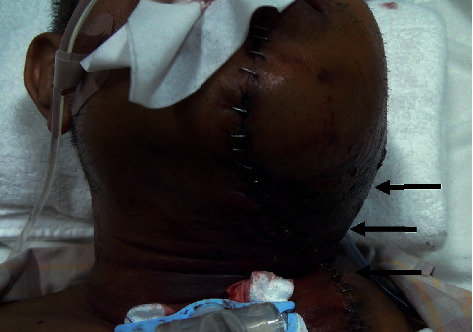
The patient's left neck swelled on the first postoperative day.

**Figure 2:**
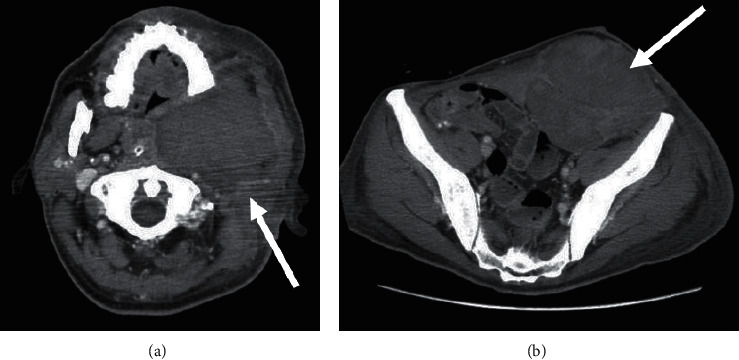
Computed tomography showed massive subcutaneous hemorrhage in the left neck (a) and abdomen (b) (arrows).

**Figure 3:**
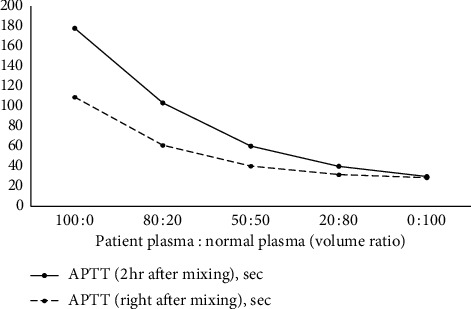
Cross-mixing test. It demonstrates no factor deficiency but suggests a delayed-type inhibitor pattern 3 days after surgery. APTT, activated partial thromboplastin time.

**Figure 4:**
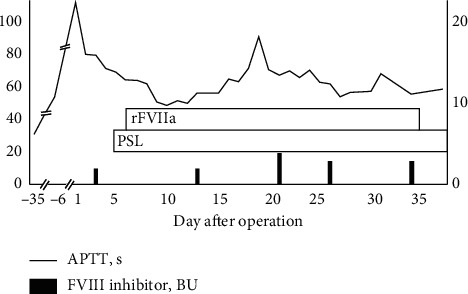
Detailed chart showing the patient's treatment progress. APTT, activated partial thromboplastin time; FVIII, coagulation factor VIII; BU, Bethesda units; rFVIIa, recombinant activated factor VIIa; PSL, prednisolone.

However, the patient's left neck and abdomen swelled on the first postoperative day (Figure 1), and computed tomography showed massive subcutaneous hemorrhage in these areas (Figure 2). Laboratory data showed a normal platelet count (201,000/mm^3^) and prolonged APTT (110.8 s), with a slightly prolonged PT-INR (1.42). The ascending pharyngeal artery was identified as the responsible vessel and embolized, using an interventional radiology approach. Red blood cells and fresh frozen plasma were transfused to treat the patient's progressive anemia. However, the bloody discharge from the cervical wounds and the extensive hematoma continued, leading us to consider the possibility of other clinical circumstances. The patient was referred to a hematology expert, and further blood coagulation tests showed that his factor II, IX, X, XI, XII, and von Willebrand factor levels were all within the normal ranges. Cross-mixing test demonstrated no factor deficiency but suggested a delayed-type inhibitor pattern (Figure 3); because, his FVIII level was remarkably low (4%), and he was positive for the FVIII inhibitor (2.0 Bethesda units (BU)). The examination was performed as an outsourced test on the third day after surgery, and we were notified of the results 5 days after surgery. On the basis of his clinical course and the laboratory data, we diagnosed AHA and recombinant activated factor VII (rFVIIa) and prednisolone 1 mg/kg/day were started. The bleeding finally stopped 35 days after the initiation of this therapy, and the subcutaneous hematoma eventually disappeared. A detailed chart of the patient's treatment progress is shown in Figure 4. The prednisolone dose was tapered and continued, but the FVIII inhibitor titer remained detectable (1.0 BU) after 8 months.

Unfortunately, local recurrence and mediastinal lymph node and lung metastases appeared 9 months postoperatively, and the patient died of cancer 3 months later.

## 3. Discussion

We report an extremely rare case of AHA presenting as massive bleeding immediately after surgery in a patient with oral squamous cell carcinoma. Notably, the patient developed sustained, severe, and uncontrolled bleeding despite appropriate primary management for postoperative bleeding, but the life was saved following input from a hematology expert. A diagnosis of AHA is usually difficult because of its rarity, especially in the postoperative setting, where the manifestations are initially considered as a major complication of the surgery. In the present case, the bleeding appeared unprecedented, even in a postoperative setting, and we promptly referred the patient to a consultant hematologist. This resulted in an accurate diagnosis and successful management of the hemorrhage with rFVIIa, as advised by the hematologist.

Most patients with autoantibodies against FVIII develop hemorrhages in their skin, muscles, and soft tissues or mucous membranes [8]. Other clinical diagnostic findings include significantly prolonged APTT, decreased FVIII activity, and the presence of FVIII autoantibody. In addition to early consultation with a hematology expert, it is important to perform further blood coagulation tests if unusual postoperative bleeding and an unexplained prolonged APTT are observed.

Several etiological factors associated with AHA have been reported in the literature, including chronic inflammatory disorders, autoimmune disorders, pregnancy, malignancies, multiple transfusions, and drugs [1–5, 7, 8]. In addition, approximately 10% of patients with AHA, many of whom have undergone surgery, have an underlying malignancy [9]. Surgery has also recently been recognized as a possible etiological factor of AHA [5]. The current patient had multiple risk factors associated with AHA, namely oral squamous cell carcinoma, surgery, and chemotherapy. Although it is not known if the AHA in this patient was associated with the surgery or the neoplasm, the FVIII inhibitor titer remained slightly elevated even after continuous immunosuppressive therapy, and the cancer recurred, suggesting that the cancer might have played a crucial role in the development of AHA in this patient.

A recent systematic literature review of AHA in cancer patients identified 60 cases of solid cancer associated with AHA, including only three cases of head and neck cancer [10]. Details of these three cases and the present case are shown in Table 1 [11–13]. The current case report thus represents only the fourth case of head and neck cancer associated with AHA. However, AHA occurred at various times regardless of surgery in the three other reviewed cases, and the present case is thus the first in which AHA developed immediately after surgery for head and neck cancer.

The fundamental principles of treating AHA include control of the bleeding and the initiation of immunosuppression to eradicate the FVIII inhibitor. The first-line treatment for bleeding in patients with AHA involves use of a bypassing agent, of which rFVIIa and activated prothrombin complex concentrate are the currently available options [14, 15]. Although the optimal therapeutic strategy for eradicating the FVIII inhibitor is unknown, current recommendations include immunosuppression with corticosteroids alone or corticosteroids in combination with cyclophosphamide [6]. A review of treatment outcomes in 41 AHA patients with cancer [9] found that complete responders were characterized by having early-stage cancer and low initial inhibitor titer, whereas incomplete responders with the persistent inhibitor were more likely to have a poor prognosis. In addition, approaches to treat the cancer itself, such as chemotherapy, surgery, or hormonal manipulation, led to eradication of the inhibitor in 22% of cases [9]. As noted above, we had to administer rFVIIa continuously in the current case because the inhibitor was not eradicated completely despite treatment with prednisolone, and the cancer eventually recurred 9 months after surgery. Clinicians should thus be alert to the possibility of cancer recurrence in patients with the persistent FVIII inhibitor after treatment.

## 4. Conclusion

We describe the fourth reported case of AHA associated with head and neck cancer in a patient with oral squamous cell carcinoma and massive postoperative bleeding. Unusual postoperative bleeding that fails to respond to conventional treatment, together with an unexplained isolated prolonged APTT, indicates the need for further hematological coagulation tests and collaboration with a hematology expert to ensure the early diagnosis and effective treatment of AHA.

## Figures and Tables

**Table 1:** Summary of reported cases of acquired hemophilia A with head and neck cancer.

Patient #	Age(years)/sex	Disease	Inhibitor titer (BU)	FVIII (%)	Bleeding control	Immunosuppression	Time of onset	Bleeding control outcome	Inhibitor eradication	Reference
1	73/F	Epiglottis (SCC)	8	<1	PCC	Hc, C, PLEX, IVIG	Presurgery	CR	CR	Shastri et al. [11]

2	73/M	Larynx	11	NA	NA	R, P	Postsurgery in cancer remission	CR	CR	Onitilo et al. [12]

3	37/M	Oral (SCC)	12	1	rFVIII, aPCC, rFVIIa	Mp	Day 29 after surgery	CR	NA	Chen et al. [13]

4	46/M	Oral (SCC)	2	4	rFVIIa	P	Day 1 after surgery	CR	PR	Present case

F, female; M, male; SCC, squamous cell carcinoma; BU, Bethesda units; FVIII, coagulation factor VIII; NA, not available; PCC, prothrombin complex concentrates; rFVIII, recombinant activated factor VIII; aPCC, activated prothrombin complex concentrate; rFVIIa, recombinant activated factor VII; Hc, hydrocortisone; C, cyclophosphamide; PLEX, plasma exchange; IVIG, intravenous immunoglobulin; R, rituximab; P, prednisolone; Mp, methylprednisolone; CR, complete response; PR, partial response.

## Abbreviations

AHA: Acquired hemophilia A
APTT: Activated partial thromboplastin time
BU: Bethesda units
FVIII: Coagulation factor VIII
PTINR: Prothrombin time-international normalized ratio
rFVIIa: Recombinant activated factor VII.
